# Entomo-virological surveillance followed by serological active survey of symptomatic individuals is helpful to identify hotspots of early arbovirus transmission

**DOI:** 10.3389/fpubh.2022.1024187

**Published:** 2022-10-28

**Authors:** André de Souza Leandro, Renata Defante Lopes, Caroline Amaral Martins, Robson Michael Delai, Daniel A. M. Villela, Rafael Maciel-de-Freitas

**Affiliations:** ^1^Centro de Controle de Zoonoses da Secretaria Municipal de Saúde de Foz do Iguaçu, Paraná, Brazil; ^2^Laboratório de Transmissores de Hematozoários, Instituto Oswaldo Cruz, Fiocruz, Rio de Janeiro, Brazil; ^3^The Instituto Latino-Americano de Economia, Sociedade e Política, Universidade Federal Latino-Americana, Foz do Iguaçu, Brazil; ^4^One Health Laboratory at the Three-Border Tropical Medicine Center, Itaiguapy Foundation - Institute of Teaching and Research, Foz do Iguaçu, Brazil; ^5^Programa de Computação Científica, Fiocruz, Rio de Janeiro, Brazil; ^6^Department of Arbovirology, Bernhard Nocht Institute for Tropical Medicine, Hamburg, Germany

**Keywords:** entomo-virological surveillance, dengue, chikungunya, early-warning system, *Aedes aegpyti*, vector-borne diseases, vector control, transmission risk

## Abstract

Arboviruses transmitted by *Aedes aegypti* in urban environments have spread rapidly worldwide, causing great impacts on public health. The development of reliable and timely alert signals is among the most important steps in designing accurate surveillance systems for vector-borne diseases. In July and September 2017, we conducted a pilot study to improve an existing integrated surveillance system by using entomo-virological surveillance to prioritize areas to conduct active searches for individuals with arbovirus infection symptoms. Foz do Iguaçu City has a permanent entomo-virological surveillance system with approximately 3,500 traps to capture *Aedes* sp. in the adult stage. The *Aedes aegypti* females are captured alive and human samples are submitted to RT-qPCR (real-time qPCR) screening for DENV, ZIKV, and CHIKV diagnosis. Of the 55 *Ae. aegypti* mosquitoes tested in July 2017, seven (12.7%) were considered positive for DENV-2 and three (5.4%) for CHIKV. In September, we tested a sample of 54 mosquitoes, and 15 (27.7%) were considered infected by DENV-2. We created 25 circumferences with 150-m radius each to perform an active survey to identify symptomatic householders. In July, we selected one circumference, and five (35.7%) patients were positive for DENV, whereas two (14.3%) for CHIKV. In September, we selected four circumferences, and, from the 21 individuals sampled, nine (42.8%) were positive for DENV-2. A statistical model with a binomial response was used to estimate the number of cases in areas without active surveys, i.e., 20 circumferences. We estimated an additional 83 symptomatic patients (95% CI: 45–145) to be found in active searches, with 38 (95% CI: 18–72) of them confirming arbovirus infection. Arbovirus detection and serotyping in mosquitoes, but also in symptomatic individuals during active surveys, can provide an alert signal of early arbovirus transmission.

## Introduction

Mosquitoes of the genus *Aedes* sp. are considered the main vectors of arboviruses in the urban environment. Among them, the special concern should be given to *Aedes aegypti*, the primary vector for dengue (DENV), Zika (ZIKV), and Chikungunya (CHIKV) in several countries worldwide. Its prominent role is due to habits that make it a species close to humans: females are preferentially anthropophilic and lay their eggs in artificial breeding sites located in the surroundings of human dwellings (1–3).

The incidence of dengue fever around the world has increased dramatically in recent decades (4). More recently, two other arboviruses transmitted by *Ae. aegypti* have been spreading rapidly: CHIKV was first detected in Brazil in 2014 after being diagnosed in the Caribbean, while ZIKV was introduced to the Northeastern Region probably the same year (5, 6). The Zika outbreak has caused a worldwide commotion, in particular, due to developmental changes in neonates, such as neurological disorders and microcephaly in newborns whose mothers were infected with ZIKV during pregnancy (7, 8).

Despite remarkable efforts from the government, public health sectors, research institutes, private companies, and non-profit organizations, achieving effective and sustainable control of *Ae. aegypti* has proven challenging. Therefore, the success of avoiding or disrupting arbovirus outbreaks has been limited (9–11). The causes are manifold but include (a) unplanned urbanization, (b) lack of basic sanitation and regular distribution of piped water, (c) greater mobility of potentially infected hosts, (d) low investment in proactive vector control measures, (e) persistent use of vector control methods that have limited efficacy, and (f) lack of timely and effective surveillance to support local decision-makers (9, 12–17).

For example, regarding arbovirus surveillance, predicting arbovirus outbreaks in time and space is challenging for public health services. If successful, it would be possible to identify hot areas of transmission and direct vector control interventions toward those regions (18–20). However, in metropolitan endemic areas, the routine monitoring of arboviruses is usually unable to detect the early stages of transmission. Thus, the absence of early-warning signs results in a dengue outbreak occasionally noticed only after an explosion of cases in the human population, causing a great impact on the primary care system and the city health network. To avoid such a scenario, elaborating timely alert signals is critical, i.e., information regarding *Ae. aegypti* infestation and arbovirus notification over the territory must be available as soon as possible to trigger appropriate responses (21, 22). Considering arboviruses' epidemiology, particularly, the extrinsic incubation period of dengue and chikungunya in *Ae. aegypti* mosquitoes, information on infected mosquitoes or infected humans should be available in 2–4 days for CHIKV (23) and 10–14 days for DENV (24).

The use of traps to capture adult mosquitoes can generate infestation indices that have greater predictive power than larval indices (25). Theoretically, the search for natural infection in insects captured alive in these traps could provide information regarding viral circulation in the mosquito population before the first human cases are reported in the city health network (18, 26–28). Thus, we hypothesized that the identification of circulating viruses in mosquitoes captured in adult traps homogeneously distributed over the city could become an indicator of the locations where the first cases of arboviruses are expected. To test this hypothesis, we used the local molecular biology facility for screening for arboviruses in the trapped insects, followed by a serological survey of suspected cases of dengue/chikungunya in the vicinity of the collection site of DENV/CHIKV-positive insects. The results of the active search and the speed of confirmation of infection in mosquitoes and humans are reported in this manuscript.

## Materials and methods

### Study area

The city of Foz do Iguaçu (25° 32' 52” S; 54° 35' 17” W) is located in the Southern Region of Brazil, State of Paraná, on the triple border with Paraguay and Argentina. The climate is well characterized by hot summers (average temperature of 33°C from December to February), cold winters (mean temperatures ranging below 11°C), and an average annual precipitation of 1,800 mm. It has around 265,000 inhabitants in 90,000 households, an urbanization rate of over 99%, and presents a high Human Development Index. The city is divided into 73 geographically distinct areas. This study occurred in the months of July and September 2017 and sought to identify human cases of dengue and chikungunya through serological surveys of symptomatic humans in areas containing trapped *Ae. aegypti* positive for either DENV or CHIKV. The choice of July and September 2017 was based on factors such as the low availability of field personnel to collect blood from symptomatic individuals and the convenience for sampling in traditional low-infestation months.

### Epidemiological surveillance: Routine

**Figure 1:**
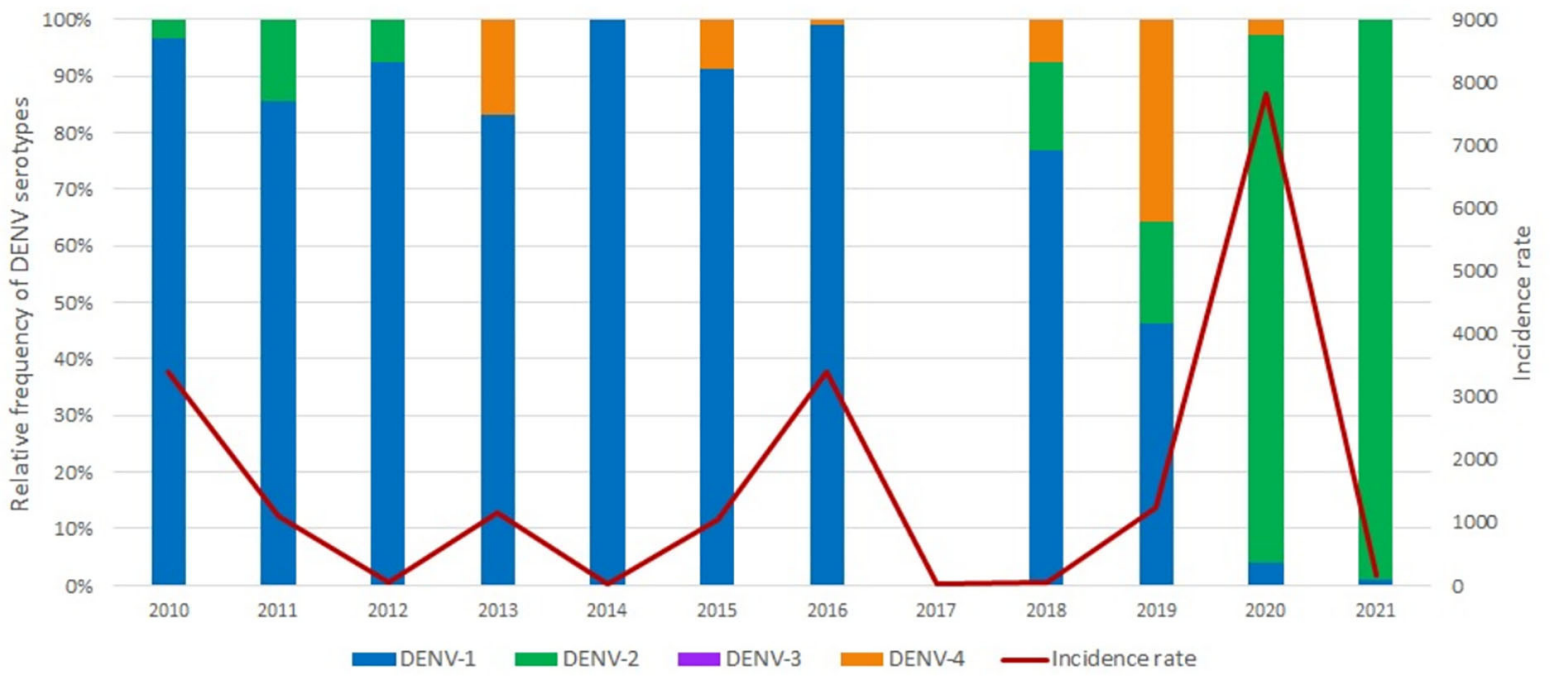
Historical data of the relative frequency of dengue serotypes and dengue incidence rate in the city of Foz do Iguacu, between 2010 and 2021.

In Brazil, arbovirus notification is predominantly passive, with symptomatic individuals seeking care in the health system. When arbovirus infection is suspected, there is an obligation to report *via* SINAN, a nationwide web-based system. An average fraction of ~40% of Foz do Iguaçu patients has their blood collected for further diagnosis by ELISA/IgM or RT-qPCR for DENV, ZIKV, and CHIKV. The diagnosis is carried out at the LACEN-PR State Central Laboratory located in Curitiba, Paraná (25° 25′ 42″ S; 49° 16′ 24″ W), approximately 650 km from Foz do Iguaçu. Between 2010 and 2021, 99,708 people sought assistance in the health system and were thus notified. Arbovirus screening was conducted in 40,495 (40.6%) people, with a total of 16,605 (41%) of them testing positive for dengue by ELISA/IgM or RT-qPCR. The average time between notification and releasing the diagnostic for DENV serotype identification on the samples has continuously decreased over time: from 149 days in 2013 to 9 days in 2021 (Figure 1).

### Entomological surveillance

Since January 2017, an integrated surveillance system has been executed in Foz do Iguaçu. The activities in the city's urban territory consist of permanent entomological surveillance by capturing adult *Ae. aegypti* traps, entomo-virological surveillance, and integration of epidemiological, demographic, and climate data in a single database (18). The adult mosquito trapping is carried out every 2 months by inspecting approximately 3,500 adults distributed throughout the city (29, 30). The traps are permanently installed in the same premises, at a ratio of one trap for every 25 houses. This trap is originally designed to collect adult *Ae. aegypti* females during oviposition, using water as its principal attractant. Water remains confined in the trap and cannot be reached by trapped mosquitoes, avoiding egg-laying. Adultraps do not become a breeding site because female *Aedes aegypti* do not lay eggs in the water within the trap. Mosquitoes are trapped after entering through a large hole on the top and becoming trapped in an interior chamber (29, 30). Historically, between 10 and 15% of the Adultraps are positive for *Ae. aegypti*, and each positive trap has an average of 2–3.8 adult *Ae. aegypti* females, depending on the collection period (18).

### Entomo-Virological surveillance

The entomo-virological surveillance consists of screening through RT-qPCR all *Ae. aegypti* mosquitoes trapped alive for the arboviruses Zika, chikungunya, and dengue, including serotype identification for the latter (31). Briefly, we extracted viral RNA from *Ae. aegypti* mosquitoes by using the MagMAX Viral/Pathogen Nucleic Acid Ultra Isolation Kit, according to the manufacturer's instructions. We added single mosquitoes individually to electromagnetic mixing beads and macerated them using TissueLyser II. For arboviral genome amplification, we used the commercial ZDC Biomol Kit (32, 33), which by a multiplex PCR reaction enables the identification of ZIKV, CHIKV, and differentiation of DENV serotypes using as positive controls positive samples from human-infected patients and an internal control (IC) of the reaction that uses probes specific to each molecular target. For PCR, we used a 96-well QuantStudio 7 Flex Real-Time PCR System and QuantStudioDesign and Analysis Software versions 1.3.1 and 1.5.1. We considered samples positive when the amplification plot curve exceeded the specific threshold for each target <35-cycle threshold. Results from the entomo-virological surveillance are available in less than 36 h after mosquitoes are collected in field traps (18).

### Epidemiological surveillance: Active surveillance for the symptomatic

In the week following the entomological surveillance, i.e., immediately after the entomo-virological surveillance, we outlined a circumference with a 150-m radius with Quantum Gis (QGIS—version 2.18). The circumference was centered in the trap, where at least one *Ae. aegypti* mosquito was naturally infected with DENV or CHIKV. The active search for symptomatic individuals took place in properties within a 150-m radius, covering an area of approximately 1,480 m^2^. A fraction of the outlined circumferences were randomly selected for active surveys, considering personnel limitations for collecting blood from symptomatic individuals. Health professionals collected whole blood samples from humans with arbovirus symptoms. A suspected (symptomatic) case of dengue fever was defined as any individual who presented one or more compatible symptoms (fever, headache, myalgia, arthralgia, rash, nausea, vomiting, retro-orbital pain, petechiae, malaise, pruritus, or conjunctivitis) in the last 14 days. The samples collected were sent for diagnosis by Real-Time PCR in the LACEN-PR. Human cases with symptoms were reported to the official surveillance system (Sistema de Informação de Agravo de Notificação—SINAN).

### Statistical analysis

Field data were stored in a PostgreSQL database, and a geographic information system was developed with the software Quantum GIS (QGIS). A statistical model in which the number of human cases in the active search was the response given a detection probability. Traps with active searches were observations analyzed in this model in which the number of cases found in the active search was a proportion *p* from the total number *T* given by the sum of cases in the active search and already counted in the usual surveillance system. The number of individuals found in the active search is given by a binomial distribution:


$$\begin{array}{l} y_{act}\;\sim \;Binomial\;\left(p,\;T\right), \end{array}$$

where the positive ratio of samples with arbovirus detection in human samples was given by parameter *q*. The number found as positives is given by a binomial distribution:


$$\begin{array}{l} y_{pos}\;\sim \;Binomial\;\left(q,\;y_{act}\right), \end{array}$$

where parameters *p* and *q* were given by log(*p/*(*1–p*) = *a* and log(*q/*(*1–q*)) = *b*, where *a* and *b* had normal prior distributions with mean 0 and precision 0.01. The number of cases in other areas without active search is estimated from prediction, given the detection probability and the number counted in the surveillance system. The number of confirmed cases is estimated as a fraction, given the probability *q*.

Parameters *p* and *q* are estimated using MCMC simulations with four chains. The total to be found in other traps is estimated as a function of the total already notified and parameter *p*, and the positive samples from parameter *q*.

### Ethical considerations

All activities regarding vector-borne disease mitigation are performed by the health agents of the Centro de Controle de Zoonoses da Secretaria Municipal de Saúde de Foz do Iguaçu (CCZ-Foz do Iguaçu). Among the actions carried out by health agents in their routine activities is the blood collection of symptomatic householders found in active surveys according to the Brazilian Program for Dengue Control guidelines, i.e., no prior submission to an Ethical Committee was required. As householders, we refer to any person responsible for a house, e.g., the owner or a tenant. In July and September 2017, a door-to-door survey for symptomatic individuals was conducted in the area within the 150-m radius centered in the trap with at least one DENV- or CHIKV-infected *Ae. aegypti*. We collected the blood of adults over the age of 18 years who gave their consent orally.

## Results

### Epidemiological surveillance: Routine

The data gathered by the epidemiological surveillance team at the LACEN-PR allow the evaluation of the relative frequency of DENV serotypes between 2010 and 2022 (Figure 1). Remarkably, there is no data available regarding DENV serotype in the Central Laboratory for the year 2017, and an annual incidence rate of 26.2 DENV cases per 100,000 inhabitants. From the total of 1921 DENV notifications registered in 2017, March and June presented the upper and lower number of cases with 232 and 68 notifications, respectively. In July and September, the months we conducted our pilot study, 81 and 226 cases were reported. In 2020, the city of Foz do Iguaçu faced its most severe DENV outbreak, with an incidence of 7,780.5 per 100,000 inhabitants (Figure 1).

### Entomological and entomo-virological surveillance

**Figure 2:**
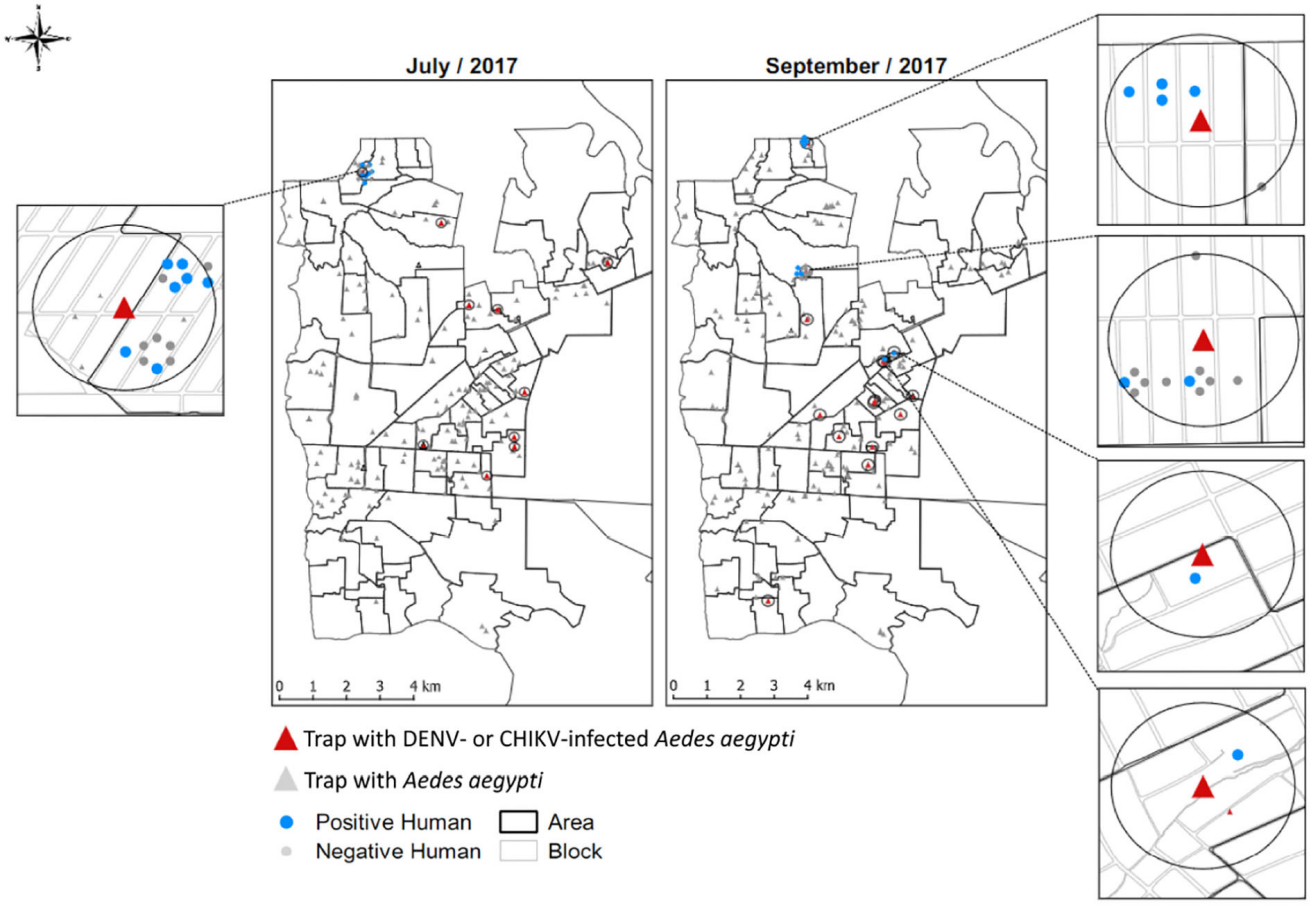
Foz do Iguaçu city map divided into 73 areas, showing the location of Adultraps with *Aedes aegypti* and those later classified as DENV- or CHIKV-infected. Highlighted in the zoomed maps is the distribution of positive and negative householders after active surveys.

In July 2017, the average infestation of Foz do Iguaçu by *Ae. aegypti* according to the Trap Positivity Index (TPI) was 7.78%. In this activity, from the 270 positive Adultraps distributed over the city, 55 of them (20.37%) at least one *Ae. aegypti* female that was captured alive and submitted to RT-q PCR screening for arbovirus. Of the samples tested, 7 (12.7%) were considered positive for DENV and 3 (5.45%) for CHIKV. Two months later, in September 2017, the average *Ae. aegypti* infestation was 6.29%. From the 54 *Ae. aegypti* females captured alive, 15 (27.7%) were considered DENV-infected (Figure 2).

### Epidemiological surveillance

In 2017, 1,921 notifications of symptomatic dengue and 29 of chikungunya were made. In July, as a pilot study, we randomly selected one of the 10 mosquito circumferences infected with arbovirus (7 for DENV and 3 for CHIKV) to conduct an active search for arbovirus-symptomatic patients. A total of 14 symptomatic individuals were identified, from whom a health professional collected whole blood samples. Of the 14 symptomatic patients, 5 were positive for DENV-2 and 2 were positive for CHIKV in the real-time PCR exam (Figure 1).

In September, we selected 4 out of 15 circumferences of 150 m centered from *Ae. aegypti* infected with DENV-2. The active survey resulted in the identification of 21 suspected human cases; 9 (42.8%) of them were positive for DENV-2 (Figure 1).

### Estimations from active search

Our estimates indicate a potential number of 83 symptomatic patients (95% CI: 45–145) in areas without an active search. The estimated number of confirmed cases in those areas is 38 (95% CI: 18–72). Overall, the number of results found in active search was almost certainly 1.2 times greater (Table 1).

**Table 1:** Observations per trap and estimations from the model.

**Observation**	**Trap**	**Month**	**Notified**	**Active search**	**Positive samples from active search**
1	1-06086	July	0	14	7
2	1-08391	September	1	5	4
3	4-06361	September	5	14	3
4	6-06582	September	5	1	1
5	6-06587	September	6	1	1
Total			17	35	16
Estimated probability in active search	0.67 (95% CrI: 0.54–0.79)				
Estimated positivity	0.46 (95% CrI: 0.30–0.62)				
	Traps with positive samples for mosquitoes		38	83 (95% CrI: 45–145)	38 (95% CrI: 18–72)

## Discussion

One of the most critical aspects of arbovirus epidemiology in endemic settings is the elaboration of alert signals to identify the early transmission of serotypes that have not been circulating recently in the region (18, 21, 22). Dengue outbreaks are highly associated with the low herd immunity of the local human population to a specific serotype that has been absent from that region in recent years (34). In the specific case of Foz do Iguaçu, we showed that (a) conducting active surveys following the entomo-virological surveillance could provide better estimates of dengue transmission by identifying symptomatic householders not seeking for assistance in local health units, (b) blood screening from sampled householders allowed us to early identify the circulation of DENV-2 in Foz do Iguaçu in 2017, an information only available by LACEN-PR on the following year, (c) the time required for identifying circulating arbovirus in mosquitoes (> 36 h in local facility) is much lower than in human samples (9 days in the LACEN-PR State Central Laboratory located in Curitiba, Paraná). Remarkably, one of the most severe dengue outbreaks in Brazil was recorded in 2020, caused mostly by DENV-2. However, CHIKV-infected mosquitoes were collected in September 2017, and so far no CHIKV outbreak has been detected in Foz do Iguaçu. This is a pattern somehow different from the rest of the country, where CHIKV outbreaks are strongly associated with their arrival in a city in which they have not circulated before (35, 36).

To our knowledge, this is the first report showing how entomo-virological surveillance can be used to trigger an active survey centered on the trap where infected *Ae. aegypti* was collected to support further identification of symptomatic householders that are not recorded by local public health.

The city of Foz do Iguaçu has started the implementation of a one health approach to improve vector-borne diseases surveillance in 2009. The adopted approach is based on 5 axes: (1) merging sectorized field teams; (2) adoption of digital solutions; (3) health agent empowerment and permanent capacity building; (4) social mobilization; and (5) active surveys (37). This manuscript belongs to the area of active survey. In Brazil, passive arbovirus surveillance is widespread, with symptomatic individuals seeking care in local health units with further compulsory notification in the nationwide web-based system called SINAN. Herein, we showed in the Foz do Iguacu context how active surveys could be incorporated into a passive surveillance system to improve epidemiological knowledge on a rapidly expanding basis.

Runge-Ranzinger et al. (22) reviewed different approaches to dengue disease surveillance and were able to group other studies according to the purpose of the surveillance system studied. They observe an increasing interest in early outbreak detection in endemic countries and assembled studies into four groups: (i) outbreak prediction or detection; (ii) monitoring dengue trends; (iii) outbreak prediction and trend monitoring; and (iv) non-endemic countries. Among the activities conducted by those interested in outbreak prediction (groups I and iii) is using the detection of a new dengue serotype as an alert signal for dengue outbreaks (38–43). Studies conducted in Singapore, Indonesia, and the Pacific Region relied on virus surveillance information using genotyped or sequenced data or hospital data and sought a correlation with the number of reported cases or dengue incidence. In Singapore, a serotype switch from DENV-2 to DENV-1 in 2004/2005 was associated with the 2005 epidemic (38, 40). Similarly, surveys in Indonesia revealed that an increase in case numbers in 2010 was attributed to a genotype shift in DENV-1 from genotype IV to I in 2009 (42). Finally, in the Pacific region, the rapid replacement of DENV-1 by DENV-4 was associated with dengue outbreaks in 2008 and 2009 in Kiribati, New Caledonia, Samoa, Tonga, and other islands (43). Notably, none of the reports used active surveillance to early detect circulating arbovirus serotypes/genotypes. In Foz do Iguaçu, the first detection of DENV-2 was in 2017 and was accomplished by screening field-caught *Ae. aegypti* and subsequently by active surveillance in an area within 150-m radius of the trap from which the infected mosquito was caught. Foz do Iguaçu faced an outbreak of DENV-2 not immediately after the following transmission season but 2 years after this shift from DENV-1 to DENV-2. The 2020 DENV-2 outbreak is the highest observed in the city since the 1990s when dengue was detected for the first time in the city. Likely, the under-notification of dengue (exemplified by finding 16 positive householders for DENV and CHIKV) played a significant role in masking the DENV-2 circulation between 2018 and 2019.

The cornerstone of the actual arbovirus surveillance in Foz do Iguaçu involves visiting 3,500 adult mosquito traps distributed over the city and screening the *Ae. aegypti* females captured alive for arboviruses (ZIKV, CHIKV, and DENV-1,−2,−3, and−4). Assuming the active surveys triggered after an infected mosquito is collected can be an alert signal for further arbovirus outbreaks, special concern should be given to actual data. So far, the Foz do Iguaçu City has not been through a chikungunya outbreak; therefore, an intensification of vector control is recommended to avoid a rapid increase in cases in the next mosquito season, traditionally from November to May.

We set the radius of 150 m centered on the collection of an arbovirus-infected *Ae. aegypti*. This rationale is supported by the average dispersal of *Ae. aegypti* females from their breeding site (44, 45). However, there are some reports in the literature showing that in particular circumstances, like in the absence of geographical barriers, *Ae. aegypti* females are capable of presenting a longer flight range (46). The definition of the ring size to perform active surveys involves a balance between literature review and manpower to conduct the surveys, and it will be further investigated under the Foz do Iguaçu settings.

The potential for finding other cases with active search strategies is shown as the number to be found can be potentially much higher than the totals that are routinely registered by regular surveillance. Such estimates may differ at other times of the year, so future analyses may benefit from additional observations and treatment based on the epidemiological scenario. As an example, between 2017 and 2020, the local arbovirus surveillance detected a total of 72 pools of *Ae. aegypti* females in which at least one individual was infected with DENV, ZIKV, or CHIKV. Low personnel availability and scarce financial support have limited the number of active surveys to a short period of time on a convenient sampling strategy. Furthermore, again due to the limited personnel availability, our study design did not consider control sites, i.e., 150-m circumferences outlined using traps with uninfected mosquitoes to estimate the likely number of potential human infections. Thus, our estimates must be taken carefully. Hopefully, our findings in regard to active surveys in a circumference with 150-m radius centered on the traps where arbovirus-infected *Ae. aegypti* females were collected could stimulate local public health managers to incorporate this activity into the epidemiological surveillance routine.

## Data availability statement

The original contributions presented in the study are included in the article/supplementary material, further inquiries can be directed to the corresponding author.

## Ethics statement

Ethical review and approval was not required for the study on human participants in accordance with the local legislation and institutional requirements. Written informed consent for participation was not required for this study in accordance with the national legislation and the institutional requirements.

## Author contributions

LA, CA, RL, and RM-d-F contributed to conception and design of the study. LA, CA, and RL organized the database. RD performed the molecular diagnostics. DV performed the statistical analysis. LA and RM-d-F wrote the first draft of the manuscript. CA, RL, DV, and RD wrote sections of the manuscript. All authors contributed to manuscript revision, read, and approved the submitted version.

## Conflict of interest

The authors declare that the research was conducted in the absence of any commercial or financial relationships that could be construed as a potential conflict of interest.

## Publisher's note

All claims expressed in this article are solely those of the authors and do not necessarily represent those of their affiliated organizations, or those of the publisher, the editors and the reviewers. Any product that may be evaluated in this article, or claim that may be made by its manufacturer, is not guaranteed or endorsed by the publisher.
